# Study on the Canine Adenovirus Type 1 (CAdV-1) Infection in Domestic Dogs in Southern Italy

**DOI:** 10.3390/pathogens11111254

**Published:** 2022-10-28

**Authors:** Francesco Mira, Roberto Puleio, Giorgia Schirò, Lucia Condorelli, Santina Di Bella, Gabriele Chiaramonte, Giuseppa Purpari, Vincenza Cannella, Andrea Balboni, Vincenzo Randazzo, Francesco Antoci, Domenico Vicari, Annalisa Guercio

**Affiliations:** 1 Istituto Zooprofilattico Sperimentale della Sicilia “A. Mirri”, Via Gino Marinuzzi n. 3, 90129 Palermo, Italy; 2 Department of Veterinary Medical Sciences, Alma Mater Studiorum—University of Bologna, Ozzano dell’Emilia, 40064 Bologna, Italy

**Keywords:** dogs, canine adenovirus, *Canine mastadenovirus A*, Italy, canine enteric viruses, infectious disease

## Abstract

Canine adenovirus type 1 (CAdV-1) is the causative agent of a systemic and potentially fatal viral disease of domestic and wild canids. In Italy, CAdV-1 infection has also been occasionally described in dogs, but information on the epidemiology and its genomic features is still limited. A study was conducted on 291 dogs suspected of infectious gastrointestinal disease. Samples collected from dogs in southern Italy between 2017 and 2020 were analyzed. Virological and histopathological assays were carried out. The presence of CAdVs and other canine viral enteropathogens was investigated, and sequence and phylogenetic analyses were performed. CAdV-1 was detected in six (2.1%) dead stray dogs alone or in mixed infections with other viruses. Gross lesions and histopathological findings referred to CAdV infection were observed, also involving the central nervous system tissues. All inoculated samples were successfully isolated. Sequence analysis evidenced divergences with the circulating strains previously described in Italy and a closer relation with older CAdV-1 strains collected from other countries, suggesting a genetic heterogeneity of CAdV-1 in Italy. The evidence of the circulation of CAdV-1 and its genomic features allows us to have more in-depth knowledge of the epidemiology and evolution of the CAdV-1 genomic variants.

## 1. Introduction

Canine adenovirus type 1 (CAdV-1) is the causative agent of a systemic and potentially fatal viral disease, nowadays uncommonly described in dogs, so far defined also as infectious canine hepatitis (ICH). ICH was first described in dogs from Sweden by the veterinary scientist Carl Swen Rubarth in the 1940s [1] and then worldwide reported in the following years also as “Rubarth’s disease” [2,3].

CAdV-1 is a non-enveloped icosahedral double-stranded DNA virus and belongs to the genus *Mastadenovirus* of the family *Adenoviridae* [4]. According to the International Committee on Taxonomy of Viruses (ICTV) (https://talk.ictvonline.org/taxonomy/, accessed on June 2022), the *Canine adenovirus* species has been recently taxonomically renamed as *Canine mastadenovirus A* [5]. CAdV-1 is genetically and antigenically closely related to the canine adenovirus type 2 (CAdV-2), the causative agent of a canine upper respiratory disease formerly known as “canine infectious tracheobronchitis”, used since after 1980 as a vaccinal strain to avoid CAdV type 1-induced vaccine-associated adverse events [6,7].

To date, CAdV-1 infection has been described worldwide both in domestic and wild animals: in *Canidae* (dogs, foxes, and wolves), *Ursidae*, and in *Mustelidae* families [8,9,10,11,12,13,14,15]. Although vaccination has reduced the circulation of CAdVs in the domestic canine population, isolated outbreaks of CAdV-1 infection have been occasionally but constantly reported, raising continuous questions about its current circulation, prevalence, and on its role as a pathogen in the domestic/wild animal interface, also including the potential risks for the endangered wild species [16,17,18,19,20]. In domestic dogs, CAdV-1 infection has been related to the low vaccination coverage in kenneled dogs or to the uncontrolled importation of dogs [9] and has been occasionally reported alone or in co-infection with other microbiological pathogens [21,22,23,24,25]. In Italy, CAdV-1 infection in dogs has also been occasionally described [17,26,27,28,29], but the information on the epidemiology and the genomic features of the circulating CAdV-1 strains is still limited.

The aim of this study was to evaluate the CAdV-1 infection in domestic dogs with signs of suspected infectious gastrointestinal disease and to provide an in-depth update on the epidemiologic, molecular, and pathogenetic features related to the CAdV-1 infection.

## 2. Materials and Methods

### 2.1. Study Design

The study was conducted on 291 dogs, showing clinical signs or anatomopathological lesions referable to infectious gastrointestinal disease. Clinical samples from live animals (rectal swabs or feces, N = 109) or carcasses of dead dogs (N = 182) were collected in Sicily region, southern Italy, between 2017 and 2020 and submitted to the Istituto Zooprofilattico Sperimentale della Sicilia “A. Mirri” (IZSSi) for diagnostic purposes.

The presence of CAdV-1 and -2, canine parvovirus type 2 (CPV-2), canine distemper virus (CDV), canine coronavirus (CCoV), and rotavirus (RoV) was investigated by molecular assays. Sequence analysis and viral isolation of the identified CAdV-1 strains and histopathological examination of the CAdV-1-positive dogs were carried out. Available data on CAdV-1-positive dogs, including signalment, anamnesis, anatomopathological, and histopathological reports, were obtained from the Informative System of Laboratories (SILAB) database of the IZSSi. Information on the vaccination status was not available.

### 2.2. Virus Screening

Tissue samples (brain, lungs, heart and pericardial fluids, thymus gland, spleen, liver, intestine, kidneys, and mesenteric and mediastinal lymph nodes) and rectal swabs/feces were collected and stored at −80 °C until use. 

Tissue samples and feces were homogenized, and supernatants were obtained, as previously described in Purpari et al. [30].

Total DNA and RNA were extracted, respectively, from 200 or 140 μL of the supernatants by using the DNeasy Blood & Tissue Kit (Qiagen S.p.A., Hilden, Germany) or the QIAmp Viral RNA Mini Kit (Qiagen S.p.A.), respectively, according to the manufacturer’s instructions. The presence of CAdV-1 and -2 DNAs was evaluated by a diagnostic PCR assay using a primer pair targeting the 3′-end fragment of the early region 3 (E3) gene and flanking regions [31]. Each amplicon was analyzed by electrophoresis on a 2% agarose gel supplemented with ethidium bromide. Extracted DNA and RNA were also amplified using a set of PCR assays for the detection of CPV-2, CDV, CCoV, and RoV, with primer pairs and probes previously described [32,33,34,35]. CPV-2 and CCoV strains from positive samples were typed according to PCR or RT-PCR assays as previously described [36,37,38]. More information on primers and probes for viral detection and typing is reported in Supplementary Material Table S1.

### 2.3. Sequence and Phylogenetic Analyses

To amplify the sequences of CAdV-1 Penton base, Hexon, and Fiber genes, three previously described primers pairs (more information in Supplementary Material Table S1) were used in separate PCR reactions [28,39]. To sequence, the partial CAdV-1 E3 gene and flanking regions (U-exon gene), CAdV-1-positive amplicons obtained in the virus screening were used.

Amplicons were purified with Illustra^TM^ GFX^TM^ PCR DNA and Gel Purification KIT (GE Healthcare Life Science, Amersham, Buckinghamshire, UK) and submitted to BMR Genomics (Padova, Italy) for direct Sanger sequencing, with a set of primers reported in Supplementary Material Table S1. Sequences were assembled according to an overlapping strategy using BioEdit ver 7.2.5 software [40] and analyzed with nBLAST (https://blast.ncbi.nlm.nih.gov/Blast.cgi, accessed in June 2022) [41] to search related sequences into GenBank database. 

Phylogenetic analysis of single (Penton base, Hexon, E3 and flanking regions, and Fiber) or concatenated (Hexon and Fiber) genes sequences was conducted with the MEGA X software [42], using the maximum-likelihood method based on the best-fit models of nucleotide substitution (Penton and Hexon: Kimura 2-parameter; E3 and Fiber: Hasegawa-Kishino-Yano (HKY); concatenated Hexon and Fiber: HKY with invariant sites). The robustness of individual nodes on the phylogeny was estimated using 1000 bootstrap replicates, and bootstrap values were indicated at the corresponding node.

### 2.4. Virus Isolation

In order to isolate the CAdV-1 strains circulating in Italy, samples were processed for viral isolation in cell cultures. Samples, processed as described above, were inoculated on confluent Madin-Darby Canine Kidney (MDCK) cell monolayers and left in contact for 30 min at 37 °C and 5% CO_2_. Culture medium, EMEM supplemented with an antibiotic and antimycotic solution (100 U/mL penicillin G sodium salt, 0.1 mg/mL streptomycin sulfate, 0.25 μg/mL amphotericin B; EuroClone^®^, Milan, Italy), was then added and the incubation was performed at 37 °C and 5% CO_2_ for 6–7 days. Inoculated cells were monitored daily, and according to standard laboratory procedures, two additional blind passages were carried out before considering virus isolation as unsuccessful. Viral growth was evaluated by the detection of cytopathic effect (CPE). Moreover, monolayers were subjected to three cycles of freeze-thawing, centrifuged at 1500× *g* for 15 min at 4 °C, and the collected supernatants were tested for CAdV DNA by PCR [31] and direct immunofluorescence assays. 

For the direct immunofluorescence assay, each sample was first inoculated on a glass chamber slide with MDCK cells using the culture medium. Incubation was performed at 37 °C and 5% CO_2_, monitored daily for 1–4 days. Thus, the medium was eliminated, the chamber was detached, and the slide was dried. The slide was fixed with acetone at −20 °C for at least 30 min and then stained using specific monoclonal antibodies against CAdV (VMRD, Inc, Pullman, WA, USA) in accordance with the manufacturer’s instructions. After washing with phosphate-buffered saline (PBS), the MDCK cells were examined under a fluorescence microscope at 200× magnification. Cells showing nuclear fluorescence were considered positive for CAdV.

Penton base, Hexon, E3 and flanking regions, and Fiber genes nucleotide sequences of the CAdV-1 isolates were obtained as described above and compared with those obtained from the tissue samples. 

CPV-2-positive samples from the CAdV-positive dogs were also processed for viral isolation in cell culture, as previously described [36]. Isolates of CAdV-1 and CPV-2 from this study were long-term stored at the Biobanca del Mediterraneo (www.bbmed.it, 20 August 2022), a biobank for the safe storage of biological material.

### 2.5. Histopathological Examination

Histopathological assays were performed according to standard procedures, and samples were stained with hematoxylin and eosin. Samples of the brain, liver, lung, spleen, lymph nodes, kidney, and intestine were immediately fixed in 10% buffered formalin for histopathological investigation.

Briefly, 4 μm thick sections were obtained by formalin-fixed paraffin-embedded tissue that were set on slides treated with silane (3-aminopropyl-trieossi-silane) in order to avoid detachment during staining. The preparations obtained were dried overnight in an oven at 37 °C. It was proceeded with dewaxing by xylene for 20 min. After a descending alcohol series (100°, 95°, 75°, and 50°), slides were washed in distilled water and then stained with hematoxylin and eosin. This was followed by the ascending scale of alcohols (50°, 75°, 95°, and 100°) and clarification in xylene. After this phase, the slides were mounted in an Acrylic mounting medium (Eukitt^®^, O. Kindler GmbH).

Immunohistochemistry was performed on samples selected for specific detection of CAdV-1 and CPV-2 viral proteins. Serial sections on glass slides were washed in xylene and hydrated in different (decreasing) concentrations of alcohol. After dewaxing, the slides were heated in a solution of sodium citrate (pH 6.0) at 96 °C for 20 min for antigen retrieval in the case of CPV primary antibody; while in the case of CAdV-1 primary antibody, proteinase K was used. Endogenous peroxidase activity was blocked with 3% hydrogen peroxide in methanol for 30 min. Slides were treated with 1% bovine serum albumin (BSA) for 30 min and incubated for 1 h at room temperature in the presence of 0.1% BSA with the primary antibodies CPV MAb IgG2a isotype (VMRD), diluted 1:200 in 0.01 M PBS and CAdV-1 MAb IgG1 isotype (VMRD), diluted 1:300. After repeated washings in PBS, the slides were incubated with secondary antibody (MACH 1 Universal HRP Polymer detection, Biocare Medical), and then incubated with DAB for colorimetric development, counterstained with Carazzi’s emallume and mounted with a coverslip using Eukitt^®^.

Two negative controls were used: the first one corresponded to a section of the positive tissue where the primary antibody was replaced by PBS; the second control, a small intestine section from a dog, was negative for CPV-2, where a primary antibody was applied. Images were acquired using a bright-light microscope, digital camera, and image capture software (Leica DMLB microscope, a Nikon DS-Fi-1 digital camera, NIS Basic Research Nikon software).

## 3. Results

### 3.1. Detection of CAdV and Co-Infections

From the 291 examined dogs, the selected viral pathogens were detected in 191 (66%) alone or in co-infection (Supplementary Material Table S2). These viruses were detected from the rectal swabs/feces of 89/109 (82%) live dogs and tissue samples of 102/182 (56%) dead dogs. CPV-2 was the most frequently detected virus (in 185/291 dogs; 63.6%), alone (from 161/185 dogs; 87%) or with other viruses (from 24/185 dogs; 13%), followed by CCoV (from 26/291 dogs; 8.9%). More details are reported in Supplementary Material Table S2. 

CAdV-1 was detected in 6/291 (2.1%) tested dogs, all among dead dogs (6/182; 3.3%), identified with numbers from 1 to 6 in Table 1. CAdV-1 was detected alone from one dog in 2020 (id. 6) and in co-infection with other viruses from one dog in 2017 (id. 1) and four dogs in 2019 (id. 2-5), respectively (Table 1). Among mixed infections, CAdV-1 was detected together with CPV-2 (one with CPV-2a, one with CPV-2b, and two with CPV-2c variants) and with CCoV subtype IIa (CCoV-IIa) in five dogs. CAdV-2 DNA was not detected in the study population.

**Figure 1:**
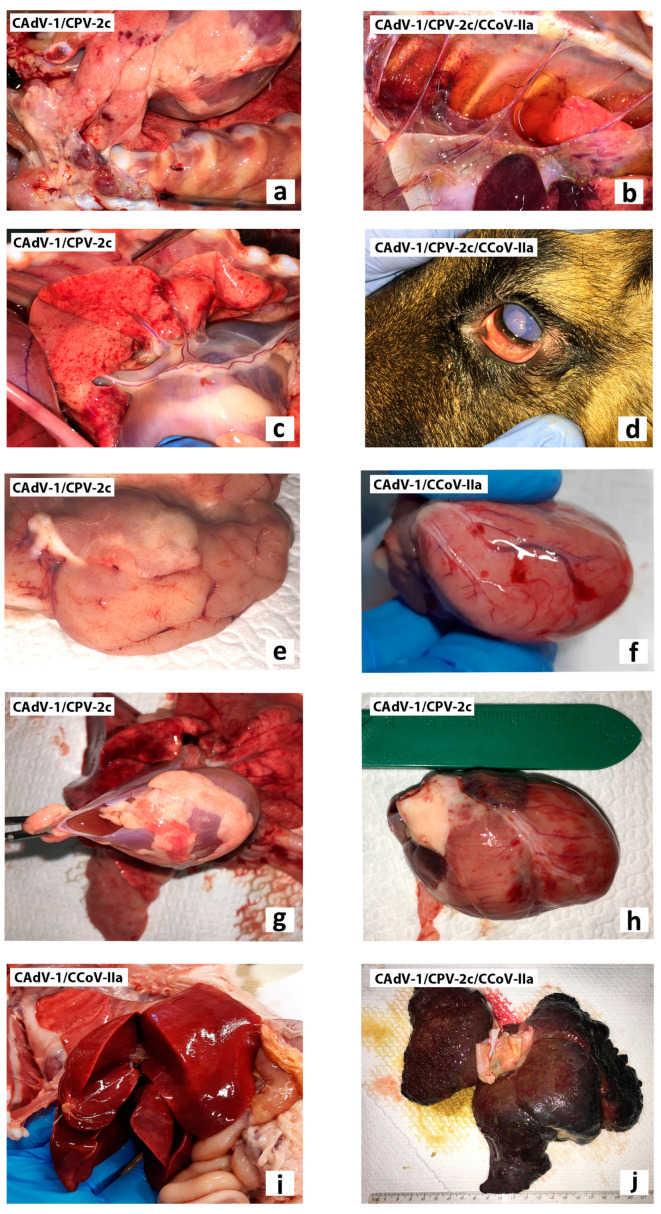
Gross lesions observed at necropsy: (**a**) ecchymosis and petechiae in thymus gland and cranial sternal lymph nodes (dog id.1, CAdV-1/CPV-2c); (**b**) moderate serous fluids and adhesions in the thoracic cavity (dog id.3, CAdV-1/CPV-2c/CCoV-IIa); (**c**) pulmonary edema with petechiae and catarrhal exudates (dog id.1, CAdV-1/CPV-2c); (**d**) corneal edema (dog id.3, CAdV-1/CPV-2c/CCoV-IIa); (**e**) brain edema and petechiae (dog id.1, CAdV-1/CPV-2c); (**f**,**h**) ecchymosis in the heart (dog id.5, CAdV-1/CCoV-IIa; dog id.1, CAdV-1/CPV-2c) and (**g**) serosanguineous fluids in pericardial cavity (dog id.1, CAdV-1/CPV-2c); enlargement and (**i**) congestion (dog id.5, CAdV-1/CCoV-IIa) or (**j**) cirrhosis (dog id.3, CAdV-1/CPV-2c/CCoV-IIa) of the liver.

All these dogs that tested positive for CAdV-1 were mixed-breed dogs, aged between two months and one year, admitted from stray to the municipal dog pounds or rescue shelters for necessary care. No information referring to the immunization of these dogs against CAdV was available. At necropsy, serosanguineous fluids in thoracic, pericardial, and abdominal cavities, ecchymoses and petechiae in several organs, pulmonary edema with catarrhal exudates, enlarged and congested liver were the main gross lesions observed, along with enteritis and enlargement of mesenteric lymph nodes (Figure 1). Details of the anatomopathological, virological, and histopathological findings of the CAdV-1-positive dogs are resumed in Supplementary Material Table S3.

### 3.2. Sequence Analysis

For the sequence analysis, single bands of the expected size referred to as the Penton base (1491 bp), Hexon (2813 bp), a fragment of the E3 and flanking regions (509 bp), and Fiber (1788 bp) genes were obtained from tissues of the six CAdV-1-positive dogs: from spleen (dogs id. 1 and 4), lung (dogs id. 2 and 3), liver (dog id. 5), and from brain (dog it. 6) samples. 

Comparison of the obtained sequences revealed a high reciprocal nucleotide (nt) identity (Penton: 100–99.86%; Hexon: 100–99.92%; E3: 100–99.60%; Fiber: 100–99.77%) between the detected CAdV-1 strains. The highest nt identity was observed for the CAdV-1 strains identified in dogs id. 2 and 3, while the lowest identities were observed for CAdV-1 from dogs id. 4 and 5. 

Comparing the CAdV-1 identified in this study with sequences available in GenBank database, Penton gene of all strains showed the highest identity rate (99.86%) with CAdV-1 ITL2015 (Italy; GenBank Acc.nr. KX545420) and CLL (Vaccine strain; U55001); Hexon gene showed the highest identity rates (100–99.92%) with CAdV-1 D43 (Japan; LC557010); E3 gene and flanking regions showed the highest identity rates (100–99.60%) with CAdV-1 D43, Wolf/835/2015/FRA (France; MH048659), GLAXO (Canada; M60937), 13-0067 (Australia; KT853097), RI261 (United Kingdom; Y07760), as well as (100–99.56%) with all sequences of CAdV-1 collected from dogs, wolves and foxes in Italy from 1966 to 2015; Fiber gene showed the highest identity rates (99.87–99.77%) with CAdV-1 D43, Wolf/835/2015/FRA, and ITL2015. 

Comparison with related CAdV-1 gene sequences retrieved from the GenBank database and, particularly, with those of CAdV-1 collected in Italy showed several nt changes resulting in synonymous and non-synonymous substitutions (Table 2). Most of these nucleotide and amino acid substitutions differentiated the CAdV-1 strains identified in this study from those previously collected in Italy, in particular, nucleotide substitutions at amino acid residues 182 and 388 in the Hexon gene, 136 and 27 in the E3 gene and U-exon region, respectively, and 23, 110, 388, and 487 in the Fiber gene.

**Figure 2:**
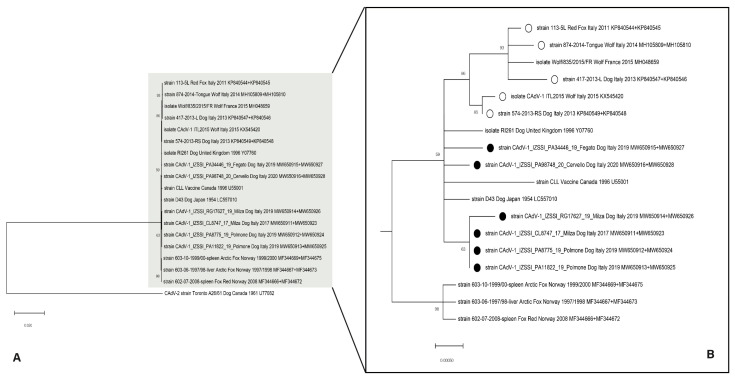
Maximum-likelihood tree (**A**) based on 18 concatenated full-length Hexon and Fiber gene sequences of canine adenovirus 1 and CAdV-2 strain sequence as outgroup (bootstrap 1000 replicates; bootstrap values greater than 60 are shown). To better draft the phylogenetic relationships between the CAdV-1 strains, the branches highlighted in grey were drawn to scale (**B**). Black dots markings (●) indicate CAdV-1 strains analyzed in this study; white dots markings (○) indicate other CAdV-1 strains previously collected in Italy. Scale bar indicates the relative number of substitutions per site. Each sequence is indicated with strain/isolate name, host, country and year of collection, and accession number.

The phylogenetic trees inferred from the single Penton, Hexon, E3 and flanking regions, and Fiber (Supplementary Material Figure S1) or concatenated (Hexon and Fiber) genes sequences (Figure 2) showed that the viruses detected in this study grouped with the CAdV-1 retrieved into the GenBank database. Due to the limited number of available sequences, the few genomic differences among them, and the low bootstrap values, the analyzed sequences resulted intermingled in the CAdV-1 cluster, without a clear and significative tree topology, but separated from the branches, including the Hexon and Fiber sequences of the strains previously collected in Italy from dogs and wild carnivores.

### 3.3. Virus Isolation

**Figure 3:**
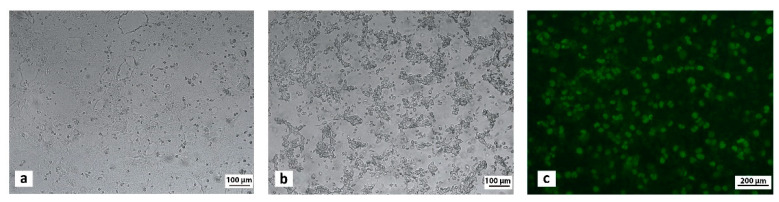
Cytopathic effect induced by CAdV-1 (**b**) on Madin Darby canine kidney (MDCK) cell monolayer (**a**) 72 h post-infection at 100× magnification. Immunofluorescence staining of MDCK cells infected with CAdV-1 at 200× magnification (**c**).

All inoculated samples induced cytopathic effect (CPE) at the second/third passage in MDCK cells (Figure 3a). CPE included typical cell rounding, clustering and detachment of the monolayer from the flask surface (Figure 3b). The presence of CAdV-1 DNA in cell cultures showing CPE was confirmed by a PCR assay. Moreover, specific fluorescence was observed in the nucleus of MDCK cells stained with the monoclonal antibody against CAdV (Figure 3c). Sequences of the CAdV-1 isolates were identical to those obtained from the samples of origin.

### 3.4. Histopathological Examination

Histology and immunohistochemistry were performed on tissues of three dead dogs that tested positive for CAdV-1 and CPV-2 viral detection (dogs id. 1,3; id. 6 only CAdV-1). 

**Figure 4:**
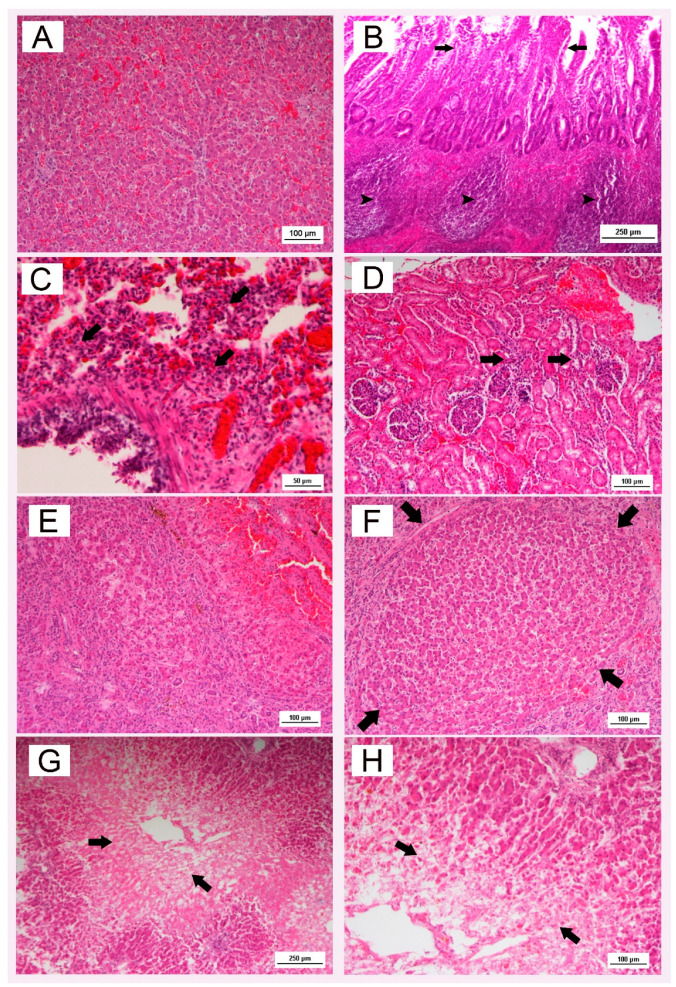
Haematoxylin and eosin stain. (**A**): Liver with reactive hepatitis; (**B**): Enteritis with marked lesions in the vicinity of Peyer’s patches (arrowheads); the mucosa was eroded and villi fused (arrows); (**C**): Lung with hemorrhage, edema, fibrin formation, and alveolar septal thickening by mononuclear cells (arrows); (**D**): Kidney with focal interstitial nephritis (arrows); (**E**): Liver with chronic hepatitis; (**F**): Liver: fibrosis, disrupting hepatic lobular architecture and culminating in the development of cirrhosis (arrows); (**G**): Hepatic centrilobular necrosis (arrows); (**H**): High magnification of centrilobular necrosis (arrows). Scale bar 100 µm; in (**C**) scale bar 50 µm; in (**B**,**G**) scale bar 250 µm.

In dog id. 1, reactive hepatitis, associated with inflammation, was observed. The histologic lesion was characterized by dissociation and rounding up of hepatocytes, accompanied by a rare infiltrate of lymphocytes (Figure 4A). In the intestinal tract, crypts were dilated and lined by cuboidal or more severely attenuated cells. The lamina propria between crypts contains numerous neutrophils, also observed in the lumen with epithelial debris. Where cryptal damage was severe, the mucosa was eroded, with fibrin and erythrocytes (Figure 4B). Lesions of lymphoid organs consisted of lymphocytolysis in follicles and paracortical tissue in lymph nodes, splenic white pulp, and gut-associated lymphoid tissue. In the lungs, hemorrhage, edema, fibrin formation, and alveolar septal thickening by mononuclear cells were observed (Figure 4C). In the brain: endothelial cells were hyperplastic and mixed with a few lymphocytes. Focal interstitial nephritis occurred with mononuclear cellular infiltrates (Figure 4D).

In the liver of dog id. 3, chronic hepatitis was observed. It was characterized by hepatocellular necrosis with a mononuclear cell infiltrate and fibrosis associated with regenerative attempts. Bridging necrosis, with tracts of necrosis dissecting across the hepatic lobule between portal areas and central veins, was also observed. Degenerative changes affecting hepatocytes included cell swelling and apoptosis. Small groups of hepatocytes were isolated, and fibrosis reached bridge portal tracts and central veins, disrupting hepatic lobular architecture and culminating in the development of cirrhosis (Figure 4E,F).

In the liver of dog id. 6, centrilobular necrosis (Figure 4G,H) was observed. The necrotic zones, initially eccentric areas about hepatic venules, extend and link up to isolate portal units. Many of the Kupffer cells are dead, others are proliferating, and others are actively phagocytic in the removal of debris. Leukocytic reactions in the liver are mild and are directed against the necrotic tissue; mononuclear cells were present, but neutrophils, many degenerating, prevailed. 

**Figure 5:**
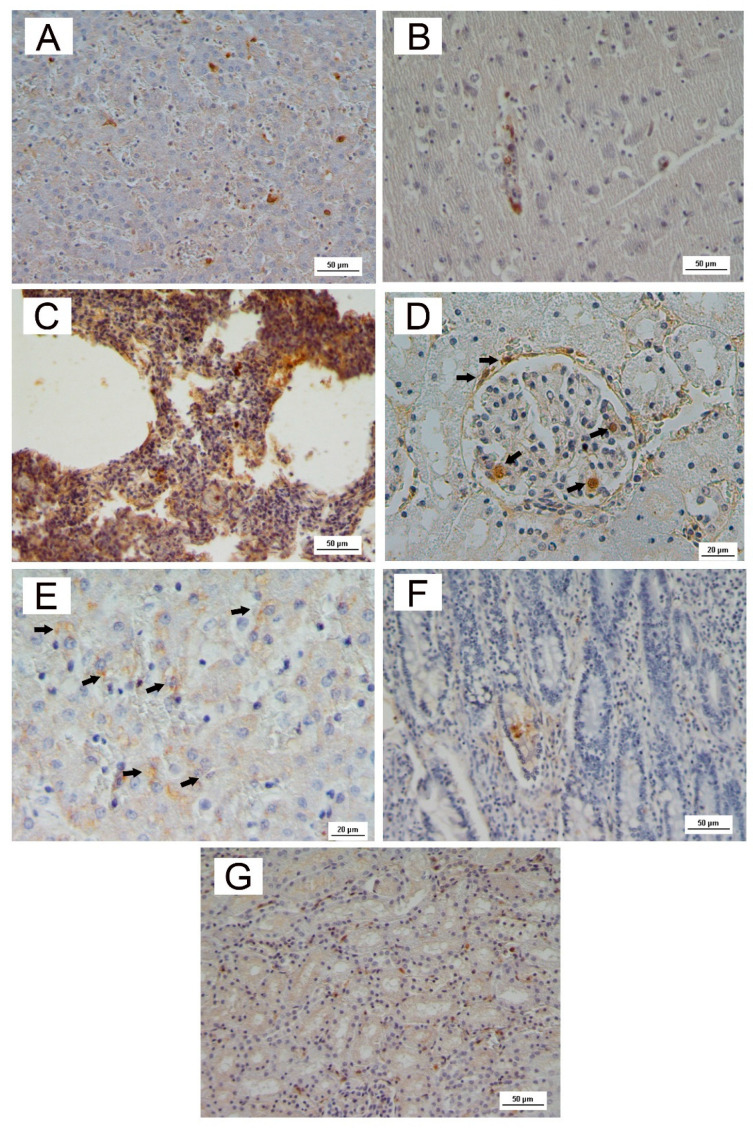
Immunohistochemistry (IHC). CAdV-1: liver (**A**); brain (**B**); lung (**C**); kidney (**D**); CPV-2: liver (**E**); intestine (**F**); kidney (**G**). Scale bar 50 µm; in (**D**,**E**), scale bar 20 µm.

Immunohistochemistry showed positive immunolabelling to antigens of CAdV-1 within hepatocytes and Kupffer cells (Figure 5A) and in endothelial cells of the brain (Figure 5B). Furthermore, antigens of CAdV-1 were observed within necrotic bronchiolar epithelial cells and peribronchiolar glandular epithelium in the dog id. 1 (Figure 5C) with interstitial pneumonia and hepatocellular disease. CAdV-1-positive cells were also observed in the kidney (Figure 5D).

Positive immunoreactivity to CPV-2 antigens was identified in the small intestine of dog id. 1 with a histopathologic diagnosis consistent with parvoviral enteritis (cryptal necrosis and dilation with fusion of villi) (Figure 5F), as also in liver and kidney (Figure 5E,G).

## 4. Discussion

In the present study, six CAdV-1 infection cases, observed through the anatomopathological and virological analyses conducted over 291 dogs sampled in the Sicily region (Southern Italy) from 2017 to 2020, were analyzed and described. This study reports the evaluation of the CAdV-1 infection by the description of the observed ICH cases through the genomic analysis of the viral strains and the histological and immunohistochemical analyses. 

For years, there was evidence of limited CAdV-1 circulation in domestic dogs and less reported was its connection to ICH cases in Italy [17,26,27,28,29,44,45]. According to these data, nowadays, CAdV-1 infection is considered uncommon in the veterinary practice, but consequentially, its clinical evidence could be underestimated. 

The most recent studies in Italy revealed a prevalence of 2.9% (4/138 dogs) of the CAdV-1 infection in dead dogs in Southern Italy in 2015–2017 [44] and of 7.8% (4/51 dogs) in alive dogs in northern Italy in 2012 [17]. All these were co-infections with CAdV-2, CPV-2, or CCoV. Similarly, our study evidenced a low frequency (6/291 dogs, 2.1%) of CAdV-1 infection in the canine population investigated, mainly associated (5/6 dogs) with CPV-2 and CCoV infections. Indeed, only one puppy (dog id. 6) tested positive exclusively for CAdV-1. All these subjects were stray dogs, and therefore, the origin of the infection was not possible to determine for each of them. Nevertheless, these data confirm the continuous circulation of CAdV-1 in the domestic canine population in the Sicily region (Italy) and, therefore, reinforce the need to consider CAdV-1 as a potential causative agent of infection and disease instead of only a neglected canine virus [17]. 

Due to the similarity of clinical signs and lesions caused by different enteric viruses as well as the potential superimposition of findings in co-infections [44,46], a wider diagnostic panel should be considered to avoid the underestimation of any of these viruses [47,48]. As observed in others, also in our study, CPV-2 remains the main causative viral agent of infection or death in dogs, mainly younger than one year of age [47,48]. Moreover, co-infections could have potentially contributed to the pathogenicity of the disease due to immunosuppressant actions and to the high rate of a negative outcome in the dogs tested in this study, as previously observed [9,23]. 

In all dogs that tested positive, CAdV-1 DNA was detected in several internal organs, accounting for a systemic infection. In the five dogs co-infected by CAdV-1 and other enteric viruses, both the anatomopathological and the histological findings reflected the infection of different viruses: the gross findings due to the systemic vascular damage (serosanguineous fluids in cavities, ecchymosis, and petechiae, enlarged and congested liver) along with the gastro-enteric lesions (catarrhal-hemorrhagic gastritis and enteritis, congestion and enlargement of mesenteric lymph nodes) could have been caused by the different pathogenetic potential of CAdV-1 and CPV-2/CCoV. Although the presence of undiagnosed causes cannot be excluded, the gross lesions and histopathological findings of the dog tested positive only for CAdV-1 (dog id. 6), revealed multifocal petechiae and a liver centrilobular necrosis, referred to the CAdV-1 infection. In two cases, petechiae due to acute severe hemorrhages were also observed in the brain surface, and this tissue tested positive for CAdV-1 DNA and CAdV-1 viral isolation. Microscopically, reactive endothelial cells with a few perivascular mononuclear cells were also observed in the brain, which tested positive for CAdV-1 immunohistochemistry. As previously observed, the endotheliotropism of the virus also includes the central nervous system (CNS) tissues, with the occurrence of acute CNS disease [9,25,29,49,50] secondary to vascular endothelial damage [51]. This evidence highlights the need for further studies to better comprehend the role of CAdV-1 in inducing neurological signs and, therefore, to be included as a differential pathogen in canine neurological syndromes. 

Sequence analysis evidenced divergences in the genome of CAdV-1 strains identified in this study compared to those of strains previously analyzed in Italy. Most of the available CAdV-1 sequences in the GenBank database are based on the partial E3 gene sequence and the 3′ flanking regions (a non-coding fragment and the U-exon gene sequences). This target, commonly described in the current literature for diagnostic assays able to distinguish between CAdV-1 and CAdV-2 [52], showed the lowest nucleotide and amino acid divergences among the tested genes. Among CAdV-1 E3 sequences obtained during the years 1966–2013 from dogs in Italy, a high sequence homology was observed, and therefore, sequences became indistinguishable based on this targeted gene. This conserved region, other than informative of the CAdV type, confirms to be a useful target both for the in-vivo and post-mortem molecular assays [17,28,44,45] but evidenced some limits for the genomic discrimination of the compared strains. Similarly, Penton base gene analysis, herein described for the first time for CAdV-1, revealed a very low divergence and rare single synonymous substitutions, preventing the evidence for genomic markers of CAdV-1 strains.

Recently, other target genes were suggested as informative to evaluate the genetic variations of this virus [20,28]. Hexon and Fiber genes encode for two major capsid proteins: the Hexon gene encodes for the proteins composing the structure of the viral capsid, the main protein targeted by the host-neutralizing antibodies, while the Fiber gene encodes for a protein that plays a role in the infection in the attaching to a specific cell surface receptor [39]. The sequence analysis of both these genes suggested a genetic heterogeneity not previously described in Italy. Indeed, according to the current literature [17,18,43] and the available sequences in the GenBank database, several amino acid residues in the deduced Hexon (182Asn, 388Asn) and Fiber (23Pro, 110Glu, 388Val, 487Val) proteins characterized the strains analyzed in this study and distinguished these strains from those previously collected in Italy from dogs (417-2013-L and 574-2013-RS), foxes (Fox/466/2017/ITA and 113-5L), and wolves (CAdV-1 ITL2015 and 874-2014-Tongue). Despite the limited number of CAdV-1 available sequences, these data highlight the recent circulation of similar but divergent strains in the Italian territory and the close genetic relation to the strains worldwide circulating in the previous decades rather than with those more recently collected from dogs or wild canids in Italy. Indeed, the contemporary presence of asparagine at position 388 in the deduced Hexon protein and of proline and glutamate at positions 23 and 110, respectively, in the deduced Fiber protein, together with additional single amino acid mutations, define a clear, typical amino acid profile [18,19], different from those of other CAdV-1 strains detected in central and northern Italy [17,18,43]. Interestingly, the CAdV-1 strains previously described in Italy were more related to a strain recently collected from a wolf in a French zoological park [19] and from a free-ranging fox cub in Apulia, Italy (authors: Prof. N. Decaro and Dr. G. Dowgier, Italy—unpublished; strain Fox/466/2017/ITA in Table 2), rather than the strains herein analyzed. Conversely, the mutations (Hexon-388Asn and Fiber-110Glu) that distinguished the strains identified in this study and matched them to older dog strains were recently reported in a wolf population in northern Canada [53], raising then new questions on the temporal and geographical spreading of these divergent CAdV-1 strains.

The limited number of studies on Hexon and Fiber gene sequences of circulating CAdV-1 strains did not allow us to clearly track the potential origin of these strains: it was not possible to determine clearly if these strains were likely already circulating in Italy or were introduced or re-introduced from other countries. Indeed, similarities were observed in both genes with a CAdV-1 strain (D43) collected in Japan from a 10-month-old spitz breed dog in 1954 [54] or with other field or vaccinal strains of the previous decades (isolate RI261; isolate CLL). Therefore, these data further raised questions about the origin of the strains described in this study, underlining the need for additional comparative studies. We could suppose that the strains herein described could be closely related to strains circulating in Italy in the previous decades [52] and, therefore, they continue to circulate in the canine population over time, but the limits in argues on their spatial-temporal tracing did not allow a definitive conclusion on their origin. Indeed, their circulation on the territory was just not previously demonstrated but could be hypothesized, and this supports the need for other further retrospective studies based on gene targets informative of these genetic variations. Nevertheless, genetic heterogeneity was observed in the Italian territory in the last decade in different geographic areas of the same country. 

For years, genetic divergences have been observed in Italy and also for other canine viruses, such as CDV [55] or CPV-2 [56]. This evidence supported the hypothesis of the co-circulation of different genetic variants of CDV and CPV-2 in the Italian territory and/or the origin from other countries or continents [55,57]. Therefore, further studies are warranted to acquire more data to clarify the origin and distribution of CAdV-1 in Italian territory, including wild animals, as well as the potential impact of the described amino acid changes.

Albeit vaccination has considerably reduced the occurrence of the CAdV-1 infection, ICH outbreaks or isolated cases continue to be periodically reported both in domestic and wild animals in Europe [19,20,28,58,59]. Vaccination against CAdV is considered a core component in canine immunization [60,61], although the reduced incidence of ICH has recently contributed to considering the CAdV component as a possible non-core vaccine, at least in some countries [7]. In Italy, CAdV is still considered a core component [62] for the correct vaccine and rational re-vaccination strategies [50,60,61].

Although national data on the vaccination of dogs are lacking, the detection of CAdV-1 infection in a background of high prevalence of CPV-2 infection suggested, at least in this area, its occurrence in a general low immune coverage and, therefore, the need for herd immunity to control or eradicate these canine viral diseases continue to be a challenge in the veterinary practice. Previous data suggested that CAdV-1 infections were related to illegal/uncontrolled canine trading or transport and/or observed in kennels or shelters [27]. In all these cases, the low vaccinal coverage was likely the fertile condition for which this and other preventable viral infections could spread [20,23]. Similarly, this condition, along with others (uncontrolled promiscuity, contact with infected animals or contaminated fomites, stressors, and co-morbidities), could affect the young stray dogs, as herein observed, and therefore, these data support the need for further control strategies and management programs of strays in urban and suburban areas other than for the dog transport or in kennels/shelters. Moreover, the current data indicate the invisible border of the domestic and wild interface, with continuous threats due to the circulation of viruses in common environments [14,15,63]. 

In conclusion, the continuous circulation of CAdV-1 and its genomic features described in this study suggest not to underestimate this virus as a causative agent of infection of domestic dogs. These results allow us to have in-depth knowledge of the epidemiology and evolution of the different CAdV-1 genetic variants for further comparative studies. 

## Figures and Tables

**Table 1:** Details on CAdV-1-positive dogs.

Dog id.	Date of Sampling	Age	Breed	Origin	Viral Co-Infections	CAdV-1 Strain	GenBank Accession Number			
							Penton	Hexon	E3	Fiber
1	19-Sep-2017	2 m	Mixed	Stray	CPV-2c	CAdV-1_IZSSI_CL8747_17_Milza	MZ313227	MW650911	MW650917	MW650923
2	01-Apr-2019	2 m	Mixed	Stray	CPV-2a	CAdV-1_IZSSI_PA8775_19_Polmone	MZ313228	MW650912	MW650918	MW650924
3	06-May-2019	12 m	Mixed	Stray	CPV-2c, CCoV-IIa	CAdV-1_IZSSI_PA11822_19_Polmone	MZ313229	MW650913	MW650919	MW650925
4	09-Oct-2019	4 m	Mixed	Stray	CPV-2b	CAdV-1_IZSSI_RG17627_19_Milza	MZ313230	MW650914	MW650920	MW650926
5	12-Dec-2019	11 m	Mixed	Stray	CCoV-IIa	CAdV-1_IZSSI_PA34446_19_Fegato	MZ313231	MW650915	MW650921	MW650927
6	17-Nov-2020	2 m	Mixed	Stray	no	CAdV-1_IZSSI_PA98748_20_Cervello	MZ313232	MW650916	MW650922	MW650928

**Table 2:** Nucleotide and deduced amino acid (in brackets) variations in Penton, Hexon, E3, U-exon, and Fiber sequences in analyzed CAdV-1 strains.

						Nucleotide and (Amino Acid) Positions															
				Penton ^a^		Hexon ^b^						E3 ^c^		U-exon ^d^	Fiber ^e^						
Strain/Isolate	Host	Country	Year	414 (138)	486 (162)	545 (182)	1128 (376)	1163 (388)	1860 (620)	2055 (685)	2238 (746)	406 (136)	427 (143)	80 (27)	69 (23)	330 (110)	513 (171)	765 (255)	1162 (388)	1460 (487)	1608 (536)
CAdV-1 ITL2015 ^1,2^	Wolf	Italy	2015	G (Ala)	T (Leu)	C (Thr)	A (Gly)	G (Ser)	T (Pro)	T (Gly)	C (Phe)	A (Ser)	T (Leu)	C (Thr)	A (Pro)	C (Asp)	T (Pro)	A (Ala)	A (Ile)	C (Ala)	C (Phe)
D43 ^1^	Dog	Japan	1954			C (Thr)	A (Gly)	A (Asn)	T (Pro)	C (Gly)	C (Phe)	A (Ser)	C (Leu)	C (Thr)	C (Pro)	A (Glu)	T (Pro)	A (Ala)	A (Ile)	C (Ala)	C (Phe)
CAdV-1_IZSSI_CL8747_17_Milza	Dog	Italy	2017					**A** **(Asn)**		**C** **(-)**			**C** **(-)**	**T** **(Ile)**	**C** **(-)**	**A** **(Glu)**				**T** **(Val)**	**T** **(-)**
CAdV-1_IZSSI_PA8775_19_Polmone	Dog	Italy	2019					**A** **(Asn)**		**C** **(-)**			**C** **(-)**		**C** **(-)**	**A** **(Glu)**				**T** **(Val)**	**T** **(-)**
CAdV-1_IZSSI_PA11822_19_Polmone	Dog	Italy	2019					**A** **(Asn)**		**C** **(-)**			**C** **(-)**		**C** **(-)**	**A** **(Glu)**				**T** **(Val)**	**T** **(-)**
CAdV-1_IZSSI_RG17627_19_Milza	Dog	Italy	2019			**A** **(Asn)**		**A** **(Asn)**	**C** **(-)**	**C** **(-)**		**G** **(Gly)**	**C** **(-)**	**T** **(Ile)**	**C** **(-)**	**A** **(Glu)**				**T** **(Val)**	**T** **(-)**
CAdV-1_IZSSI_PA34446_19_Fegato	Dog	Italy	2019	A (-)	C (-)			**A** **(Asn)**		**C** **(-)**	**T** **(-)**		**C** **(-)**		**C** **(-)**	**A** **(Glu)**		**G** **(-)**	**G** **(Val)**		
CAdV-1_IZSSI_PA98748_20_Cervello	Dog	Italy	2020				**G** **(-)**	**A** **(Asn)**		**C** **(-)**			**C** **(-)**		**C** **(-)**	**A** **(Glu)**	**C** **(-)**				
Fox/466/2017/ITA ^2^	Fox	Italy	2017	n.a. ^3^				G (Ser)		T (-)		n.a. ^3^			C (-)	C (Asp)					
874/2014 ^2^	Wolf	Italy	2014	n.a. ^3^				G (Ser)		T (-)			C (-)		C (Thr)	A (Glu)					
574-2013-RS ^2^	Dog	Italy	2013	n.a. ^3^				G (Ser)		T (-)			C (-)		C (-)	C (Asp)					
417-2013-L ^2^	Dog	Italy	2013	n.a. ^3^				G (Ser)		T (-)			C (-)		C (Thr)	A (Glu)					
113-5L ^2^	Fox	Italy	2013	n.a. ^3^				G (Ser)		T (-)			C (-)		C (Thr)	A (Glu)					
300-2012-RS ^2^	Dog	Italy	2012	n.a. ^3^		n.a. ^3^							C (-)		n.a. ^3^						
09-13F ^2^	Fox	Italy	2011	n.a. ^3^		n.a. ^3^							C (-)		n.a. ^3^						
313-2010-Lparaffin ^2^	Dog	Italy	2010	n.a. ^3^		n.a. ^3^							C (-)		n.a. ^3^						
384-1966-FFPEL ^2^	Dog	Italy	1966	n.a. ^3^		n.a. ^3^							C (-)		n.a. ^3^						

^1^ Reference strains; ^2^ Other strains collected in Italy; ^3^ Gene sequence not available. Positions are referred to the ^a^ ORF11 (12498-13931), ^b^ ORF16 (16754-19471), ^c^ ORF23 (25125-25709), ^d^ ORF24 (25718-25885), ^e^ ORF25 (25884-27515) of the reference strain CAdV-1 ITL2015 (acc.no. KX545420-reference [43]). Strain/isolate accession numbers: strain CAdV-1 ITL2015 (KX545420), D43 (LC557010), Fox/466/2017/ITA (Hexon: MH399790; Fiber: MH399791), 874/2014 (Hexon: MH105809; E3: MH105808; Fiber: MH105810), 574-2013-RS (Hexon: KP840549; E3: KP670424; Fiber: KP840548), 417-2013-L (Hexon: KP840547; E3: KP670423; Fiber: KP840546), 113-5L (Hexon: KP840545; E3: JX416839; Fiber: KP840544), 300-2012-RS (KF676980), 09-13F (JX416838), 313-2010-Lparaffin (KF676977), 384-1966-FFPEL (KP670422).

## Data Availability

Sequence data have been submitted to the GenBank databases under accession numbers MZ313227-MZ313232 (Penton gene), MW650911-MW650916 (Hexon gene), MW650917-MW650922 (E3 gene), and MW650923-MW650928 (Fiber gene).

## Supplementary Materials

The following supporting information can be downloaded at: https://www.mdpi.com/article/10.3390/pathogens11111254/s1, Figure S1: Maximum-likelihood tree and CAdV-1 clade drawn to scale based on 11 Penton (A), 20 Hexon (B), 36 E3 and flanking regions (C), and 19 Fiber (D) gene sequences, respectively, and CAdV-2 strain sequence as outgroup (bootstrap 1000 replicates). Black dots markings (●) indicate CAdV-1 strains analyzed in this study. Scale bar indicates the relative number of substitutions per site. Each sequence is indicated with strain/isolate name, host, country and year of collection, and accession number; Table S1: Primers, probe, and expected amplicons size for the viral detection, typing, and sequencing; Table S2: Number of positive dogs and frequency of detection; Table S3: Anatomopathological, virological, and histopathological findings of the CAdV-1 cases.
