# Supplementary material for: Study on the Canine Adenovirus Type 1 (CAdV-1) Infection in Domestic Dogs in Southern Italy
Source: Pathogens. 2022 Oct 28;11(11):1254. doi: 10.3390/pathogens11111254 (PMC9693395; doi:10.3390/pathogens11111254)
Supplement: Supplementary file 1 [file pathogens-11-01254-s001.zip › pathogens-1906625 - Supplementary/Supplementary Material Figure S1.pdf]

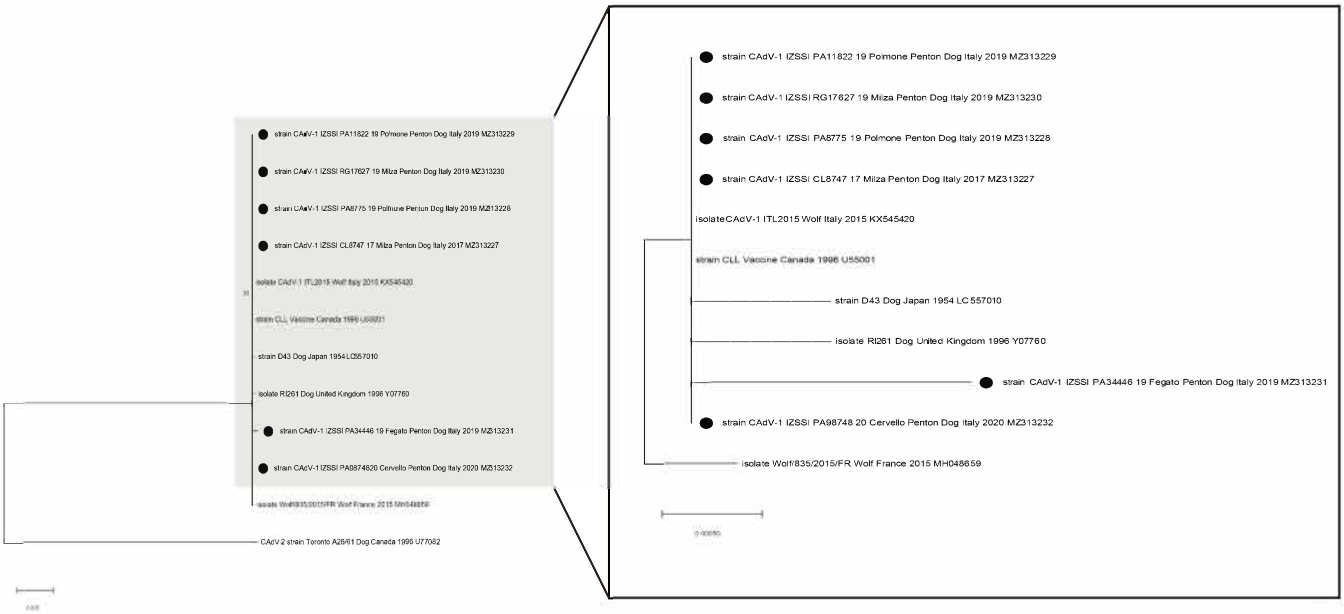
strain CAdV-1 IZSSI PA11822 19 Polmone Penton Dog Italy 2019 MZ313229
strain CAdV-1 IZSSI RG17627 19 Milza Penton Dog Italy 2019 MZ313230
strain CAdV-1 IZSSI PA8775 19 Polmone Penton Dog Italy 2019 MZ313228
strain CAdV-1 IZSSI CL8747 17 Milza Penton Dog Italy 2017 MZ313227
isolate CAdV-1 ITL2015 Wolf Italy 2015 KX545420
strain CLL Vaccine Canada 1996 U55001
strain D43 Dog Japan 1954 LC557010
isolate RI261 Dog United Kingdom 1996 Y07760
strain CAdV-1 IZSSI PA34446 19 Fegato Penton Dog Italy 2019 MZ313231
strain CAdV-1 IZSSI PA98748 20 Cervello Penton Dog Italy 2020 MZ313232
isolate Wolf/835/2015/FR Wolf France 2015 MH048659
CAdV-2 strain Toronto A26/61 Dog Canada 1996 U77082

A

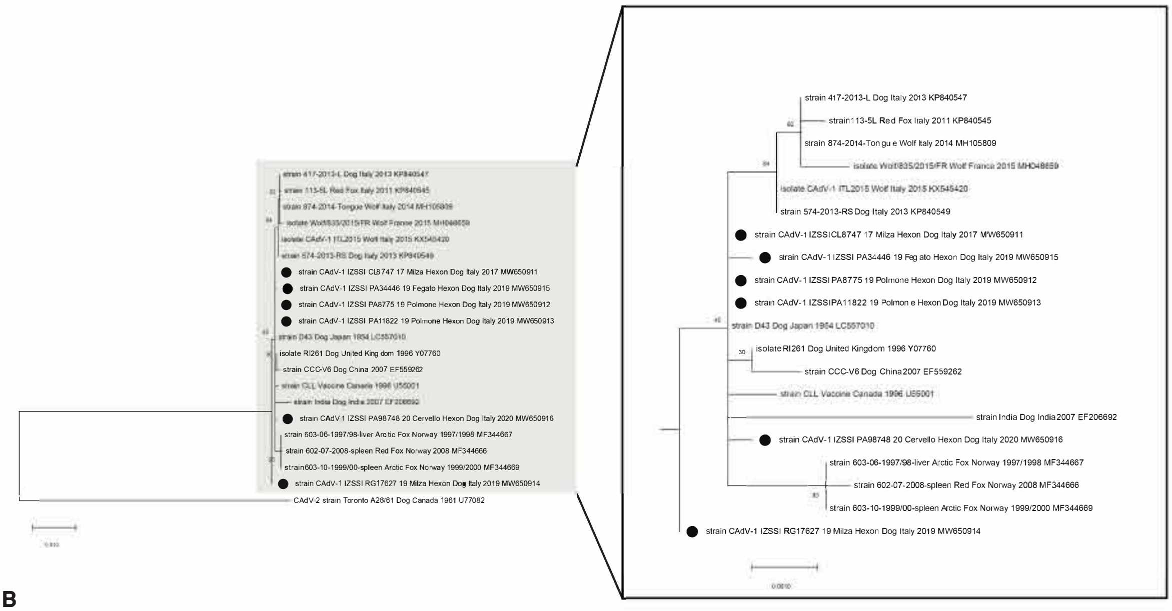
strain 417-2013-L Dog Italy 2013 KP840547
strain 113-5L Red Fox Italy 2011 KP840545
strain 874-2014-Tongue Wolf Italy 2014 MH105809
isolate Wolf/835/2015/FR Wolf France 2015 MH048659
isolate CAdV-1 ITL2015 Wolf Italy 2015 KX545420
strain 574-2013-RS Dog Italy 2013 KP840549
strain CAdV-1 IZSSI CL8747 17 Milza Hexon Dog Italy 2017 MW650911
strain CAdV-1 IZSSI PA34446 19 Fegato Hexon Dog Italy 2019 MW650915
strain CAdV-1 IZSSI PA8775 19 Polmone Hexon Dog Italy 2019 MW650912
strain CAdV-1 IZSSI PA11822 19 Polmone Hexon Dog Italy 2019 MW650913
strain D43 Dog Japan 1954 LC557010
isolate RI261 Dog United Kingdom 1996 Y07760
strain CCC-V6 Dog China 2007 EF559262
strain CLL Vaccine Canada 1996 U55001
strain India Dog India 2007 EF206692
strain CAdV-1 IZSSI PA98748 20 Cervello Hexon Dog Italy 2020 MW650916
strain 603-06-1997/98-liver Arctic Fox Norway 1997/1998 MF344667
strain 602-07-2008-spleen Red Fox Norway 2008 MF344666
strain 603-10-1999/00-spleen Arctic Fox Norway 1999/2000 MF344669
strain CAdV-1 IZSSI RG17627 19 Milza Hexon Dog Italy 2019 MW650914
CAdV-2 strain Toronto A26/61 Dog Canada 1961 U77082

B

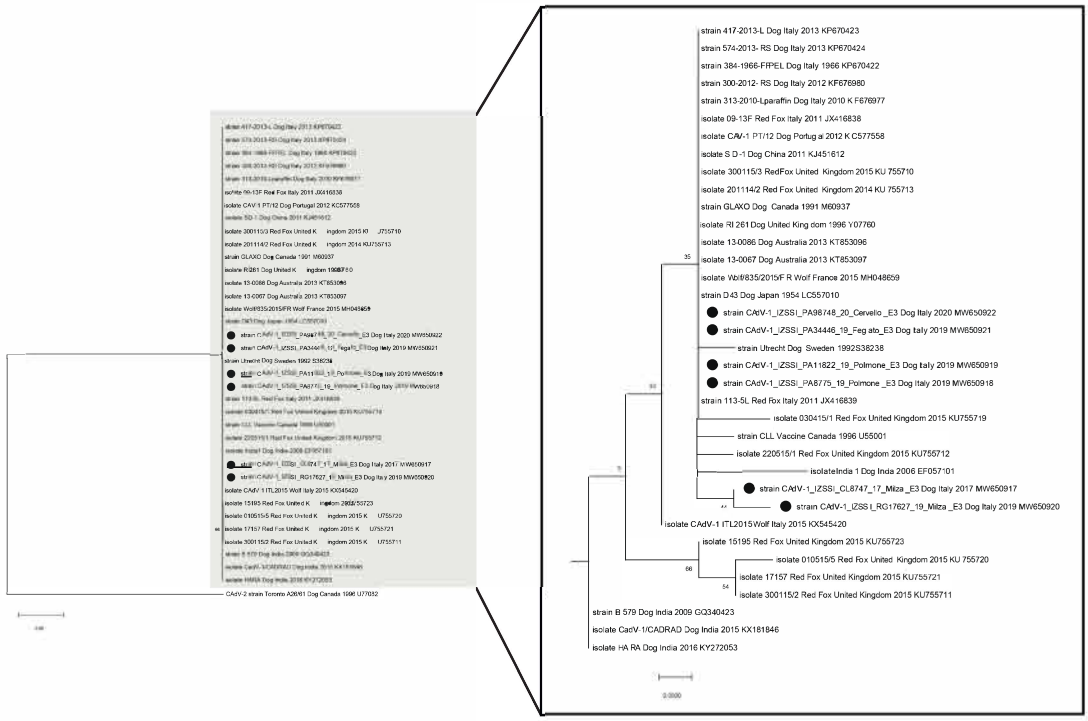

C

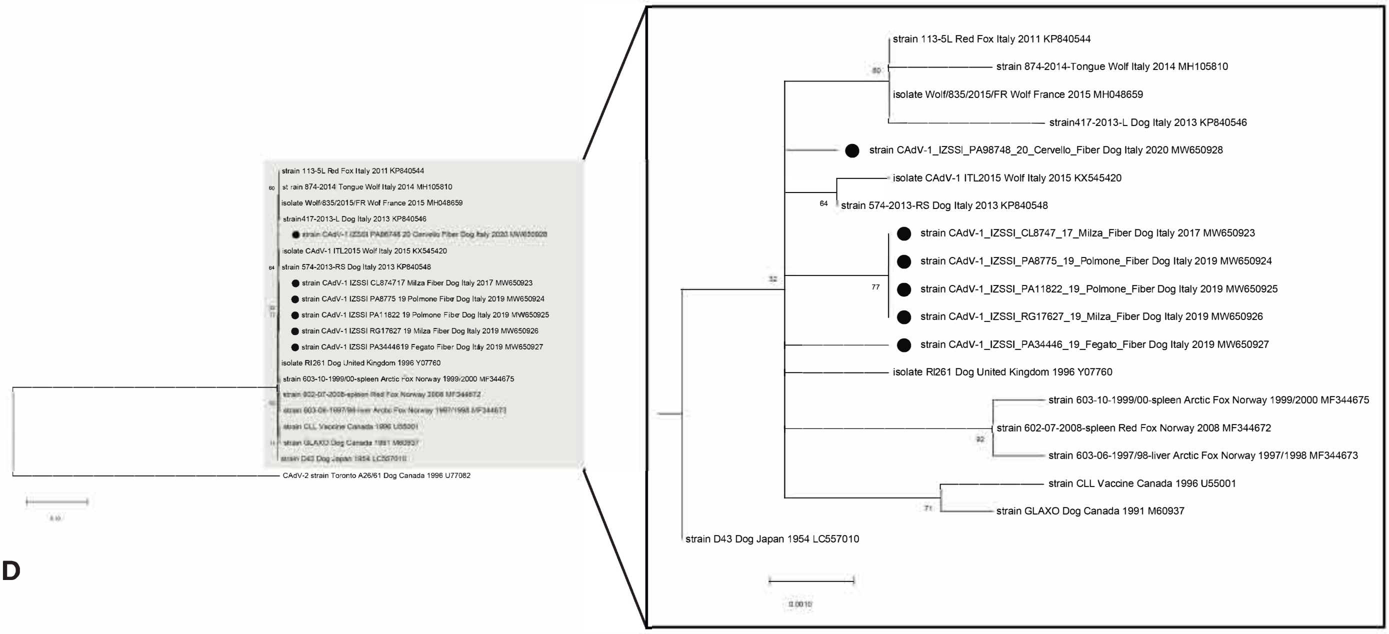

D

Supplementary Material Figure S1. Maximum-likelihood tree and CAdV-1 clade drawn to scale based on 11 Penton (A), 20 Hexon (B), 36 E3 and flanking regions (C), and 19 Fiber (D) gene sequences, respectively, and CAdV-2 strain sequence as outgroup (bootstrap 1,000 replicates). Black dots markings (●) indicate CAdV-1 strains analysed in this study. Scale bar indicate the relative number of substitutions per site. Each sequence is indicated with strain/isolate name, host, country and year of collection, and accession number.
