# Supplementary material for: Study on the Canine Adenovirus Type 1 (CAdV-1) Infection in Domestic Dogs in Southern Italy
Source: Pathogens. 2022 Oct 28;11(11):1254. doi: 10.3390/pathogens11111254 (PMC9693395; doi:10.3390/pathogens11111254)
Supplement: Supplementary file 1 [file pathogens-11-01254-s001.zip › pathogens-1906625 - Supplementary/Supplementary Material Table S1.pdf]

Supplementary Material Table S1. Primers, probe, and expected amplicons size for the viral detection, typing, and sequencing.

Virus	Target	Primer	Primer sequence (5'-3')	Amplicon size (bp)	Reference
CAdV	Early region 3 (E3) gene and flanking regions	[1]HA1	CGCGCTGAACATTACTACCTTGTC	508 (CAdV-1)	[31]
		[1]HA2	CCTAGAGCACTTCGTGTCCGCTT	1030 (CAdV-2)	
	Penton gene	[2]CAdV2-penFF	AATGGAGTTTTCGTCGTCTCCT	1491 (CAdV-1)	[39]
		[2]CAdV2-penRR	TTAGTCGGTGATATTAAGATGGC		
	Hexon gene	[2]CAdV-Hexon-For1	GAAGTTTGCCGACCCTGTC	2813	[28]
		[2]CAdV-Hexon-Rev1	ACTATGGCTCGCAGCTCTTC		
		[4]CAdV-Hexon-For2	CTGATGCCTWTAAATACACACCTGA		
		[4]CAdV-Hexon-Rev2	GACCTGAATATGGAACGAGCA		
	Fiber gene	[2]CAdV-Fiber-For1	ATGTGGTCTCTCCCRACAGC	1787 (CAdV-1)	
		[2]CAdV-Fiber-Rev1	ACTTTTCCTGAAGGCGGYAG	1748 (CAdV-2)	
		[4]CAdV-Fiber-For2	ACCATATAACTCTAAGTGCTGGGAG		
		[4]CAdV-Fiber-Rev3	TATGGGAAAGTCCAGGTCCA		
CPV-2	VP2 gene	[1]VP2-850-Forward	GAGCATTGGGCTTACCA	700	[32]
		[1]VP2-1550-Reverse	GCAAGATGCATCAGGATC		
		[2,3]P1	ATGAGTGATGGAGCAGTTC	1745	[36]
		[2,3]VPR	TTTCTAGGGTGCTAGTTGAG		
		[4]41-FOX2	GCCCTTGTGTAGACGC		
		[4]353:	CCTGGATTAAACCAAACTCC		
CDV	P gene	[1]DMV 1	ATGTTTATGATCACAGCGGT	429	[33]
		[1]DMV 2	ATTGGGTTGCACCACTTGTC		
CCoV	M gene	[1]CCV1	TCCAGATATGTAATGTTCGG	409	[34]
		[1]CCV2	TCTGTTGAGTAATCACCAGCT		
	M gene (CCoV-I)	[2]CCoV1a	GTGCTTCCTCTTGAAGGTACA	239	[37]
		[2]CCoV2	TCTGTTGAGTAATCACCAGCT		
	M gene (CCoV-II)	[2]Can1F	TAACATTGCTCTCAGGGAATTTG	202	
		[2]CCoV2	TCTGTTGAGTAATCACCAGCT		
	S gene (CCoV-I)	[2]EL1F	CAAGTTGACCGTCTTATTACTGGTAG	346	[37]
		[2]EL1R	TCATATACGTACCATTATAGCTGAAGA		
	S gene (CCoV-IIa)	[2]S5	TGCATTTGTGTCTCAGACTT	694	
		[2]S6	CCAAGGCCATTTTACATAAG		
	S gene (CCoV-IIb)	[2]CEPol-1	TCTACAATTATGGCTCTATCAC	370	[38]
		[2]TGSP-2	TAATCACCTAAMACCACATCTG		
RoV	NSP3 region	[1]NVP3-FDeg	ACCATCTWCACRTRACCCTC	87	[35]
		[1]NVP3-R1	GGTCACATAACGCCCCTATA		
		[5]NVP3-Probe	ATGAGCACAATAGTTAAAAGCTAACA CTGTCAA		

Primers used for [1]detection, [2]typing, and [3]sequencing of CAdV (canine adenovirus), CPV-2 (canine parvovirus type 2), CDV (canine distemper virus), CCoV (canine coronavirus), and RoV (rotavirus); [4]additional internal primers used for sequencing. [5]probe labeled with reporting dye (6-FAM) and with quenching dye (MGB).
