# Supplementary material for: Study on the Canine Adenovirus Type 1 (CAdV-1) Infection in Domestic Dogs in Southern Italy
Source: Pathogens. 2022 Oct 28;11(11):1254. doi: 10.3390/pathogens11111254 (PMC9693395; doi:10.3390/pathogens11111254)
Supplement: Supplementary file 1 [file pathogens-11-01254-s001.zip › pathogens-1906625 - Supplementary/Supplementary Material Table S2.pdf]

Supplementary Material Table S2. Number of positive dogs and frequency of detection.

	Single infection			Mixed infection				Total
	CPV-2	CCoV	CAdV-1	CPV-2 CCoV	CPV-2 CAdV-1	CAdV-1 CCoV	CPV-2 CAdV-1 CCoV	
Positive	161	4	1	20	3	1	1	191
Frequency (%)	84.3	2.1	0.5	10.5	1.6	0.5	0.5	
