# Supplementary material for: Study on the Canine Adenovirus Type 1 (CAdV-1) Infection in Domestic Dogs in Southern Italy
Source: Pathogens. 2022 Oct 28;11(11):1254. doi: 10.3390/pathogens11111254 (PMC9693395; doi:10.3390/pathogens11111254)
Supplement: Supplementary file 1 [file pathogens-11-01254-s001.zip › pathogens-1906625 - Supplementary/Supplementary Material Table S3.pdf]

Supplementary Material Table S3. Anatomopathological, virological, and histopathological findings of the CAdV-1 cases.

Dog id.	Date of submission	Signalment and anamnesis	Anatomopathological findings	Virological findings	Histopathological findings
1	Sep. 2017	Six mixed-breed puppies were admitted from stray at childbirth, without receiving maternal colostrum. Few days after the first vaccine administration (CDV and CPV-2 based), puppies died after showing mild gastroenteric and neurological clinical signs.	Serosanguineous fluids in pericardium, ecchymosis and petechiae in thymus gland and cranial sternal lymph nodes, heart, mesenteric lymph nodes and brain were observed. Liver appeared enlarged and congested. Pulmonary oedema with catarrhal exudates and petechiae were also evidenced.	CAdV-1 detected in the brain, pericardial fluid, heart, lung, thymus gland, spleen, liver, intestine, kidney, mediastinal and mesenteric lymph nodes.<br>CPV type 2c detected in the heart, thymus gland, spleen, kidney, intestine, mediastinal and mesenteric lymph nodes.	Mononuclear enteritis; lymphocytolysis in lymph nodes and splenic white pulp; reactive hepatitis; interstitial nephritis; interstitial pneumonia; brain: reactive endothelial cells with a few perivascular mononuclear cells.
2	Apr. 2019	A 40 days old mixed-breed puppy was admitted from stray to the municipal dog pound. Few days after the first vaccine administration (CPV-2 based), puppy showed haemorrhagic diarrhoea for 5 days. Despite the complete recovery, seven days later puppy suddenly died without apparently showing clinical signs.	Anaemia of the explorable mucous membranes, moderate serous fluids in the thoracic cavity, ecchymosis and petechiae in thymus gland and cranial sternal lymph nodes were observed. Pulmonary oedema with catarrhal exudates, mainly in the cranial lobes, were evidenced. Liver appeared enlarged and congested.	CAdV-1 detected in the lung, liver, intestine, kidney.<br>CPV type 2a detected in the heart, lung, spleen, intestine.	NO
3	May 2019	A young mixed-breed dog was admitted from stray to the municipal dog pound. Dog showed dehydration and emaciation, corneal oedema and massive ascites but, despite repeated medicaments and treatments, death occurred few days later.	Anemia of the explorable mucous membranes, moderate serous fluids and adhesions in the thoracic cavity, enlarged mediastinic lymph nodes were observed. A haemorrhagic gastritis, severe hepatic cirrhosis and enlarged mesenteric lymph nodes were also observed.	CAdV-1 detected in the lung, spleen, liver, intestine.<br>CPV type 2c detected in the brain, heart, lung, spleen, intestine.<br>CCoV-II detected in the intestine.	Chronic hepatitis; interstitial nephritis; interstitial pneumonia.
4	Oct. 2019	A four-months old mixed-breed stray puppy was found dead with the suspect of poisoning. Toxicological assays (dicumarol, warfarin, metaldehyde,	Anemia of the explorable mucous membranes and mild dehydration were observed. Bowel loops showed signs of haemorrhagic enteritis, and mesenteric lymph nodes appeared	CAdV-1 detected in the spleen and lung.<br>CPV type 2b detected in the spleen, intestine and mesenteric lymph nodes.	NO

		organophosphate and organochlorine pesticides, methomyl) tested negative.	enlarged and haemorrhagic. Lungs hyperaemia and pulmonary oedema were also observed.		
5	Dec. 2019	One year old mixed-breed dog was admitted from stray to the municipal dog pound and vaccinated (CPV-2 based). Suddenly died without apparently showing clinical signs.	Hyperaemia of the explorable mucous membranes and perineal fouling from diarrheal stools were observed. Moderate hemotorax and sero-sanguineous fluids in pericardium and abdominal cavity were also observed. Liver appeared enlarged.	CAdV-1 detected in the intestine, spleen, liver, heart, kidney and lung.<br>CCoV-II detected in the intestine and lung.	NO
6	Nov. 2020	A two-months year old mixed-breed puppy was admitted from stray to a private shelter. The dog died suddenly without apparently showing clinical signs, except for an alteration of the heart murmur	Liver appeared enlarged and congested, petechiae and ecchymosis were observed in heart and kidneys. Areas of lung atelectasis were also observed.	CAdV-1 detected in the brain, intestine, liver, kidney and lung.	Liver centrilobular necrosis.
